# The efficacy of curcumin-piperine co-supplementation on clinical symptoms, duration, severity, and inflammatory factors in COVID-19 outpatients: a randomized double-blind, placebo-controlled trial

**DOI:** 10.1186/s13063-022-06375-w

**Published:** 2022-06-06

**Authors:** Gholamreza Askari, Amirhossein Sahebkar, Davood Soleimani, Atena Mahdavi, Sahar Rafiee, Muhammed Majeed, Farzin Khorvash, Bijan Iraj, Mahshid Elyasi, Mohammad Hossein Rouhani, Mohammad Bagherniya

**Affiliations:** 1 Food Security Research Center, Isfahan University of Medical Sciences, PO Box: 00983137922110, Isfahan, Iran; 2 Anesthesia and Critical Care Research Center, Isfahan University of Medical Sciences, Isfahan, Iran; 3 Department of Community Nutrition, School of Nutrition and Food Science, Isfahan University of Medical Sciences, Isfahan, Iran; 4 Applied Biomedical Research Center, Mashhad University of Medical Sciences, Mashhad, Iran; 5 Biotechnology Research Center, Pharmaceutical Technology Institute, Mashhad University of Medical Sciences, Mashhad, Iran; 6 School of Medicine, The University of Western Australia, Perth, Australia; 7 Research Center of Oils and Fats, Kermanshah University of Medical Sciences, Kermanshah, Iran; 8 Sabinsa Corporation, East Windsor, NJ USA; 9 Nosocomial Infection Research Center, Isfahan University of Medical Sciences, Isfahan, Iran; 10 Isfahan Endocrine and Metabolism Research Center, Isfahan University of Medical Sciences, Isfahan, Iran

**Keywords:** COVID-19, Curcumin, Piperine, Clinical trial, Clinical symptoms

## Abstract

**Background:**

COVID-19 pandemic has made the disease a major global problem by creating a significant burden on health, economic, and social status. To date, there are no effective and approved medications for this disease. Curcumin as an anti-inflammatory agent can have a positive effect on the control of COVID-19 complications. This study aimed to assess the efficacy of curcumin-piperine supplementation on clinical symptoms, duration, severity, and inflammatory factors in patients with COVID-19.

**Methods:**

Forty-six outpatients with COVID-19 disease were randomly allocated to receive two capsules of curcumin-piperine; each capsule contained 500 mg curcumin plus 5 mg piperine or placebo for 14 days.

**Results:**

Mean changes in complete blood count, liver enzymes, blood glucose levels, lipid parameters, kidney function, and c-reactive protein (CRP) were not significantly different between the two groups. There was a significant improvement in health status, including dry cough, sputum cough, ague, sore throat, weakness, muscular pain, headache, and dyspnea at week 2 in both curcumin-piperine and placebo groups (*P* value < 0.05); however, the improvement in weakness was more in the curcumin-piperine group than with placebo group (*P* value 025).

**Conclusion:**

The present study results showed that curcumin-piperine co-supplementation in outpatients with COVID-19 could significantly reduce weakness. However, in this study, curcumin-piperine co-supplementation could not significantly affect the other indices, including biochemical and clinical indices.

**Trial registration:**

Iranian Registry of Clinical Trials IRCT20121216011763N46. 2020-10-31

## Introduction

The coronavirus disease 2019 (COVID-19), first recognized in Wuhan, Hubei province, China, in December 2019, was declared as a global pandemic by the World Health Organization (WHO) [1]. The infection causes mild to severe respiratory symptoms in all age groups, although the elderly and individuals with comorbidities like hypertension, diabetes mellitus, cardiovascular diseases, and obesity are at higher risk [2, 3]. The clinical manifestation of the disease starts from 2 to 14 days after getting infected and mainly involves fever, cough, weakness, and shortness of breath [2, 4]. This disease can affect personal lives and whole economies, industries, and nations [5]. The mortality related to COVID-19 is mainly caused by respiratory distress and acute respiratory distress syndrome (ARDS) [6]. High levels of cytokine including interleukin (IL)-2, IL-17 [7], interferon-γ inducible protein, monocyte chemoattractant protein (MCP)-1, macrophage inflammatory protein (MIP)1-α, granulocyte colony-stimulating factor (GSF), and tumor necrosis factor (TNF)-α contribute to ARDS [8, 9]. Oxygen therapy and other supportive care continue as the primary therapy for patients with COVID-19 pneumonia [8] for symptomatic relief.

Curcumin, usually used as the yellow spice, is obtained from the roots of the Curcuma longa [10, 11]. Curcumin is a natural polyphenolic compound, which has pleiotropic therapeutic effects and is well known for its antioxidant and anti-inflammatory activities [10, 12–14]. Curcumin is also known to decrease the expression of wide range of cytokines, which are associated with chronic inflammatory diseases [15]. In animal models, curcumin supplementation was shown to modulate inflammatory response and reduce respiratory distress [16]. Curcumin could bind to SARS-CoV-2 proteins and, thus, may have potential antiviral effects. The in silico docking studies discovered that curcumin could potentially inhibit ACE2 and spike glycoprotein of the virus to suppress COVID-19 entry to the cell [6, 10, 17]. The proven anti-inflammatory activity of curcumin could prevent cytokine [8] storms, and the molecule may also be effective in alleviating the coagulation abnormalities observed in COVID-19 patients [17–19]. Furthermore, curcumin’s [8], immunomodulatory, anti-microbial, antiviral properties, and beneficial effects of curcumin on pneumonia are evidenced [20–22].

Due to the several unique properties of curcumin and its beneficial effects on different aspects of human health, several reviews and also some clinical trials have recently suggested that curcumin might have beneficial effects on signs and symptoms of COVID-19 [6, 19, 23–27]. Results from clinical trials showed that curcumin as a natural polyphenolic compound could be a potential treatment for COVID-19 infection [19, 23, 25, 28]. A review study reported some of the potential effects of curcumin such as inhibiting the entry of viruses to the cell, inhibiting encapsulation of the virus and viral protease [6]. Another study has shown that nano-curcumin can contribute to the increased rate of inflammatory cytokines especially IL-1β and IL-6 mRNA expression and cytokine secretion in COVID-19 patients [29]. Although curcumin has low absorption, the addition of piperine improves its absorption and bioavailability of [30–32]. It is revealed that administration of piperine with curcumin resulted in 2000% increase in bioavailability of curcumin [33]. Therefore, in this study, we evaluated the effect of curcumin-piperine co supplementation on clinical symptoms, duration, severity, and inflammatory factors in outpatients with COVID-19.

## Methods and materials

### Study design and participants

This study was a parallel randomized, double-blind, placebo-controlled clinical trial evaluating the efficacy of co-supplementation of curcumin-piperine on COVID-19 outpatients. Participants were recruited from referred outpatients who had COVID-19 confirmed via real-time polymerase chain reaction (RT-PCR) at the Motamed health center, affiliated with Isfahan University of Medical Sciences, Isfahan, Iran, from November 2020 to April 2021. The summary of the study protocol, available in the Journal of Trials [34], was approved by the ethics committee of the Isfahan University of Medical Sciences, with the ethical code: IR.MUI.MED.REC.1399.049. In this study, we only reported the data obtained from COVID-19 outpatients. This trial was conducted in accordance with the principles of the Declaration of Helsinki. All patients were informed regarding the objectives and procedures of the trial, who then provided written informed consent. The trial was registered in the Iranian Registry of Clinical trials with ID: IRCT20121216011763N46.

COVID-19 patients 18 to 65 years, with a diagnosis of COVID-19 confirmed by RT-PCR, were included in the trial. Patients with severe disease admitted to the hospital were excluded from the study. Other exclusion criteria were recent use of warfarin or other anticoagulant drugs and a history of sensitivity to herbal products such as turmeric and pepper. Patients unwilling to continue participation, compliance with the trial of less than 80%, or any adverse events were withdrawn from the trial.

### Trial randomization and blinding

Eligible participants were randomly allocated in a ratio of 1:1 to either the intervention group or the control group. Randomization was stratified according to sex (male vs. female), with the use of permuted block size of 4. The assignment sequences were provided by an independent statistician with the use of a random-number table and then were kept in opaque, sealed, numbered envelopes until the end of the eligibility criteria evaluation. Treatment assignments were concealed from researchers and all patients until the completion of data analyses. In this double-blind study, capsules (curcumin and placebo) were labeled A and B by the company in the packages with the same format. Capsules were similar in terms of size, shape, color, and odor. Investigators, participants, laboratory staff, outcome assessors, and data analyzers were blinded to treatment assignment until the completion of data analyses.

### Intervention

Participants in the intervention group received two capsules of curcumin-piperine; each capsule contained 500 mg curcumin plus 5 mg piperine (totally, 1000 mg curcumin and 10 mg piperine/ day) while those in the control group received matching placebo capsules; each capsule contained 505 mg maltodextrin (totally 1010 mg maltodextrin/ day). Patients were asked to consume two capsules/day for 14 days. The capsule curcumin-piperine and matching placebo were provided by the Sami Labs Limited (Bangalore, India). In both groups, patients were asked to consume capsules at 9 and 18 o’clock for 14 days. Frequent phone calls obtained participants’ compliance (assessed by counting the remaining capsule). All patients were visited by one physician in Motamed health center. All the subjects were given standard treatment as per their physician prescriptions and allowed to take their usual medications considering the fact that adjuvant treatment alone is not ethical.

### Socio-demographic and anthropometric parameter assessment

An expert nutritionist collected socio-demographic information using a structured interview. Demographic data including age, sex, marital status, education, medications, and underlying diseases including diabetes, hypertension, and heart diseases were collected from all subjects. In addition, weight was recorded with 0.1 kg accuracy, and height was measured with 0.1 cm accuracy with Seca scales. Then, body mass index (BMI) was calculated using the following equation:

$$\mathrm{weight}\left(\mathrm{kg}\right)/{\mathrm{height}}^2\ \left(\mathrm{height}\ \mathrm{in}\ \mathrm{meters}\ \mathrm{squared}\right)$$

### Clinical outcome assessment

An internal medicine specialist visited eligible patients and evaluated the clinical signs and symptoms of COVID-19 using a structural questionnaire. This questionnaire included these items: dry cough, sputum cough, ague, sore throat, weakness, muscular pain, headache, and dyspnea. The patient graded the severity of clinical symptoms for each item as very low, low, middle, high, and very high. The improvement of clinical symptoms for each item was defined as a reduction of at least one degree from the baseline to the end of week 2 of the trial.

### Biochemical outcome assessment

Fasting blood samples (5 ml) were collected before and after the trial. The samples were centrifuged at room temperature for 10 min to isolate serum and then were immediately stored at -80°C. The enzymatic methods with auto-analyzer were used to measure complete blood count (CBC), fasting blood sugar (FBS), serum cholesterol, triglyceride (TG), low-density lipoprotein (LDL), high-density lipoprotein (HDL), very low-density lipoprotein (VLDL), alanine aminotransferase (ALT), aspartate aminotransferase (AST), lactate dehydrogenase (LDH), creatinine, blood urea nitrogen (BUN), and C-reactive protein (CRP) using commercial kits (Pars Azmun, Karaj, Iran).

### Statistical analysis

The statistical package for the social sciences (SPSS) software version 16 (SPSS Inc., Chicago, IL, USA) was used to analyze data. Within-group differences were ascertained using Paired sample t-test (normally distributed variables), Wilcoxon rank-sum test (ordinal variables and non-normally distributed variables), and chi-squared test or Fisher’s exact test (nominal variables). Also, between-group differences were ascertained using Independent Student’s t-test (normally distributed variables) and Mann-Whitney *U* test (ordinal variables and non-normally distributed variables). Data were reported as mean ± standard deviation (SD) or frequency (percentage). A *P*-value of less than 0.05 was considered to indicate statistical significance.

## Results

**Fig. 1:**
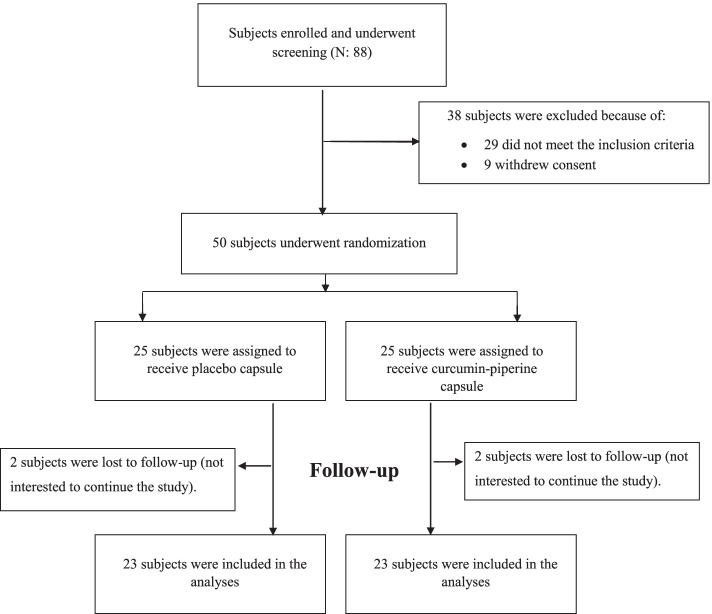
Screening, randomization, treatment, follow-up

Among 88 patients with COVID-19 disease, 50 subjects met the inclusion criteria and were included in this trial. Four patients, two in the curcumin-piperine group and two in the placebo group, discontinued the trial. Finally, 46 patients (23 in the curcumin group and 23 in the control group) completed the trial and were included in the statistical analysis (Fig. 1). No serious adverse effects were reported throughout the trial.

The mean age (SD) of patients was 47.63 (13.89) years, and the mean weight (SD) was 74.55 (16.27) kg. The demographic characteristics of participants in each group are shown in Table 1. The mean age, weight, height, and BMI did not differ significantly between the curcumin and placebo groups (*P* value >0.05). Also, the distribution of the patients in terms of gender, presence of diabetes, hypertension, heart diseases, drug usage, and levels of education was similar between the groups (*P* value >0.05).

**Table 1:** Patients’ demographic and anthropometric characteristics in the curcumin and placebo groups

Variables	Curcumin group (***N***: 23)	Placebo group (***N***: 23)	***P*** value
Male, *n* (%)	14 (60.9)	13 (56.5)	0.765^†^
Female, *n* (%)	9 (39.1)	10 (43.5)	
Married, *n* (%)	18 (78.3)	19 (82.6)	0.710^†^
Age, years	43.74 ± 12.9	51.52 ± 13.8	0.056
Weight, kg	78.94 ± 12.9	73.39 ± 12	0.141
Height, cm	170.04 ± 9.7	165.34 ± 7.8	0.077
Body mass index (BMI), kg/m^2^	26.74 ± 3.9	26.35 ± 4.7	0.763
Diabetes, *n* (%)	7 (30.4)	5 (21.7)	0.502^†^
Hypertension, *n* (%)	5 (21.7)	6 (26.1)	0.730^†^
Heart diseases, *n* (%)	4 (17.4)	3 (13)	1^††^
Academic education, *n* (%)	12 (52.2)	9 (39.1)	0.375^†^
Drug usage, *n* (%)	13 (56.5)	16 (69.6)	0.359^†^

Data are reported as mean ± standard deviation or frequency (percentage) as appropriate

^†^*P* values were calculated using the chi-squared test

^††^*P* values were calculated using the Fisher exact test

*P values were calculated using the two-sample *T* test

The mean changes in laboratory outcomes from baseline to the end of the intervention period in each group are shown in Table 2. The mean WBCs, MCH, MCHC, and total cholesterol significantly increased in the placebo group at the end of the intervention period (*P* value <0.05). In the curcumin group, the mean RDW and total cholesterol significantly increased at the end of the intervention period (*P* value <0.05). However, there were no significant differences between the curcumin group and the placebo group (Table 3). Ten patients (77%) in the curcumin group and ten patients (77%) in the placebo group recovered from dry cough, with no significant difference between the two groups. Seven patients (87.5%) in the curcumin group and six patients (85.7%) in the placebo group recovered from sputum cough, which there was no significant difference between the two groups. Four patients (66.7%) in the curcumin group and seven patients (100%) in the placebo group recovered from ague, which there was no significant difference between the two groups. Five patients (71.4%) in the curcumin group and five patients (83.3%) in the placebo group recovered from sore throat, which there was no significant difference between the two groups. Ten patients (83.3%) in the curcumin group and five patients (38.5%) in the placebo group recovered from weakness. This improvement was significantly greater in the curcumin group than in the placebo group (*P* value = 025). Seven patients (70%) in the curcumin group and seven patients (50%) in the placebo group recovered from muscular pain, which there was no significant difference between the two groups. Nine patients (69.2%) in the curcumin group and four patients (50%) in the placebo group recovered from headaches, which there was no significant difference between the two groups. Five patients (62.5%) in the curcumin group and five patients (83.3%) in the placebo group recovered from dyspnea, which there was no significant difference between the two groups.

**Table 2:** Mean changes of biochemical factors in the curcumin and placebo groups from baseline to 2-week intervention

Variables	Group	Before	After	***P*** value^†^	Mean change	***P*** value*
RBC (× 10^12^/L)	Curcumin (*N*: 23)	5.00 ± 0.62	4.98 ± 0.58	0.668	− 0.02 ± 0.19	0.404
	Placebo (*N*: 23)	4.83 ± 0.62	4.74 ± 0.48	0.246	− 0.09 ± 0.38	
WBCs (×10^9^ L)	Curcumin (*N*: 23)	6.36 ± 1.73	6.33 ± 1.49	0.94	− 0.03 ± 1.68	0.053
	Placebo (*N*: 23)	6.15 ± 1.68	7.22 ± 2.69	0.018	1.06 ± 1.97	
Hb; g/dL	Curcumin (*N*: 23)	14.47 ± 10.51	14.55 ± 10.56	0.544	0.09 ± 0.67	0.418
	Placebo (*N*: 23)	13.74 ± 11.47	14.29 ± 8.06	0.318	0.55 ± 2.59	
Hct; %	Curcumin (*N*: 23)	42.60 ± 5.38	42.76 ± 5.33	0.721	0.17 ± 2.21	0.616
	Placebo (*N*: 23)	40.72 ± 6.58	41.72 ± 4.18	0.525	1.00 ± 7.39	
MCV; fL	Curcumin (*N*: 23)	85.49 ± 8.40	84.73 ± 11.47	0.600	− 0.76 ± 6.82	0.562
	Placebo (*N*: 23)	88.38 ± 5.04	88.46 ± 5.14	0.819	0.08 ± 1.68	
MCH; pg	Curcumin (*N*: 23)	29.03 ± 3.84	28.83 ± 4.27	0.658	0.19 ± 2.06	0.172
	Placebo (*N*: 23)	29.90 ± 2.49	30.32 ± 2.64	0.003	0.42 ± 0.58	
MCHC; g/dL	Curcumin (*N*: 23)	33.86 ± 1.78	33.90 ± 1.73	0.836	0.04 ± 0.91	0.081
	Placebo (*N*: 23)	33.77 ± 7.29	34.25 ± 1.68	0.004	0.48 ± 0.72	
RDW; %	Curcumin (*N*: 23)	13.45 ± 1.34	13.68 ± 1.58	0.039	0.23 ± 0.48	0.186
	Placebo (*N*: 23)	13.41 ± 1.29	13.43 ± 1.39	0.885	0.02 ± 0.58	
PLT; 10^3^/μL	Curcumin (*N*: 23)	262.28 ± 106.8	256.42 ± 67.29	0.772	− 5.85 ± 95.71	0.296
	Placebo (*N*: 23)	203.9 ± 56.02	222.50 ± 70.61	0.150	18.55 ± 59.71	
ESR; mm/h	Curcumin (*N*: 23)	11.28 ± 11.90	11.23 ± 10.85	0.760	− 0.59 ± 9.17	0.556
	Placebo (*N*: 23)	15.69 ± 13.96	13.52 ± 9.31	0.256	− 2.17 ± 8.93	
PT; seconds	Curcumin (*N*: 23)	10.58 ± 0.58	10.90 ± 1.01	0.161	0.31 ± 1.06	0.112
	Placebo (*N*: 23)	10.87 ± 0.91	10.76 ± 0.86	0.466	− 0.12 ± 0.77	
INR	Curcumin (*N*: 23)	1.02 ± 0.05	1.05 ± 0.10	0.184	0.03 ± 0.10	0.137
	Placebo (*N*: 23)	1.05 ± 0.05	1.05 ± 0.10	0.539	− 0.01 ± 0.05	
FBS, mg/dL	Curcumin (*N*: 23)	126.52 ± 77.52	126.78 ± 61.53	0.967	0.26 ± 30.19	0.633
	Placebo (*N*: 23)	114.02 ± 42.34	109.61 ± 26.21	0.613	− 4.43 ± 41.47	
LDH, U/L	Curcumin (*N*: 23)	320.18 ± 59.23	323.28 ±72.38	0.869	2.74 ± 79.06	0.630
	Placebo (*N*: 23)	354.24 ± 72.38	345.04 ± 62.16	0.628	− 9.47 ± 91.44	
ALT, U/L	Curcumin (N: 23)	25.52 ± 16.75	25.78 ± 14.73	0.288	− 2.74 ± 12.05	0.427
	Placebo (*N*: 23)	40.26 ± 26.30	33.30 ± 18	0.146	− 6.95 ± 22.18	
AST, U/L	Curcumin (*N*: 23)	22.61 ± 8.30	22.00 ± 7.20	0.636	− 0.61 ± 6.09	0.151
	Placebo (*N*: 23)	31.78 ± 17.52	25.86 ± 6.29	0.960	− 5.92 ± 16.32	
Chol, mg/dL	Curcumin (*N*: 23)	162.08 ± 33.22	181.34 ± 43.82	0.001	19.26 ± 24.67	0.800
	Placebo (*N*: 23)	166.69 ± 38.30	188.08 ± 48.81	0.004	21.39 ± 31.53	
TG, mg/dL	Curcumin (*N*: 23)	168.61 ± 134	144.00 ± 103	0.239	− 24.61 ± 97.6	0.206
	Placebo (*N*: 23)	140.52 ± 54.7	144.57 ± 58.7	0.665	4.04 ± 44.2	
VLDL, mg/dL	Curcumin (*N*: 23)	29.85 ± 14.59	26.61 ± 15.74	0.431	− 3.09 ± 18.48	0.365
	Placebo (*N*: 23)	28.13 ± 10.80	28.82 ± 11.66	0.705	0.69 ± 8.69	
LDL, mg/dL	Curcumin (*N*: 23)	95.00 ± 26.44	100.39 ± 33.21	0.086	5.39 ± 14.35	0.409
	Placebo (*N*: 23)	98.48 ± 24.91	108.22 ± 29.57	0.333	9.74 ± 20.49	
HDL, mg/dL	Curcumin (*N*:23)	41.39 ± 8.54	41.52 ± 5.18	0.932	0.13 ± 7.39	0.345
	Placebo (*N*: 23)	38.43 ± 4.37	40.30 ± 5.47	0.068	1.87 ± 4.66	
CRP; mg/L	Curcumin (*N*: 23)	4.12 ± 2.83	3.26 ± 11.47	0.254	− 0.86 ± 3.55	0.846
	Placebo (*N*: 23)	3.54 ± 2.54	2.63 ± 8.30	0.047	− 1.04 ± 2.35	
BUN, mg/dL	Curcumin (*N*: 23)	12.04 ± 2.78	12.04 ± 2.83	0.998	0.00 ± 2.16	0.824
	Placebo (*N*: 23)	13.04 ± 3.46	13.21 ± 4.22	0.784	0.17 ± 3.02	
Creatinine mg/dL	Curcumin (*N*: 23)	0.74 ± 0.09	0.56 ± 0.53	0.043	− 0.17 ± 0.38	0.468
	Placebo (*N*: 23)	0.61 ± 0.48	0.52 ± 0.53	0.328	− 0.09 ± 0.43	
GFR, mL/min	Curcumin (*N*: 23)	70.18 ± 19.39	75.86 ± 13.44	0.147	5.68 ± 19.44	0.231
	Placebo (*N*: 23)	74.74 ± 17.52	75.26 ± 18.67	0.688	0.52 ± 6.14	

Data are reported as mean ± standard deviation

^†^*P* values were calculated using the paired sample *t* test

*P values were calculated using the two-sample *t* test

**Table 3:** Within- and between-group comparisons of the clinical outcomes from baseline to the end of the intervention

Variables	Positive cases	Clinical outcomes		***P*** value^†^	***P*** value*
		Improving	No change		
Dry cough	Curcumin group: 13	10	3	0.002	1
	Placebo group: 13	10	3	0.002	
Sputum cough	Curcumin group: 8	7	1	0.008	0.788
	Placebo group: 7	6	1	0.014	
Ague	Curcumin group: 6	4	2	0.046	0.366
	Placebo group: 7	7	0	0.008	
Sore throat	Curcumin group: 7	5	2	0.025	0.626
	Placebo group: 6	5	1	0.025	
Weakness	Curcumin group: 12	10	2	0.002	0.025
	Placebo group: 13	5	8	0.025	
Muscular pain	Curcumin group: 10	7	3	0.008	0.337
	Placebo group: 14	7	7	0.008	
Headache	Curcumin group: 13	9	4	0.003	0.390
	Placebo group: 8	4	4	0.046	
Dyspnea	Curcumin group: 8	5	3	0.025	0.441
	Placebo group: 6	5	1	0.025	

Data are reported as frequencies

^†^Within-group comparisons with the use of the Wilcoxon rank-sum test

*Between-group comparisons with the use of the Mann–Whitney *U* test

## Discussion

Currently, the COVID-19 pandemic has made the disease a major global problem by creating a significant burden on health, economic and social status [35]. On the other hand, despite the relentless efforts by researchers all over the world, no effective treatment for this disease has been found yet [36]. Natural compounds are known to have several health benefits and may prove valuable as an adjunct therapy for this infection by reducing the severity and morbidity of the disease. Consequently, this study was performed to evaluate the efficacy of curcumin-piperine co-supplementation on COVID-19.

The most important outcome of the present study was a significant reduction in weakness due to curcumin-piperine co-supplementation. However, no significant difference was observed between the intervention and placebo groups in the other clinical symptoms and laboratory factors. However, findings of the present study are in contradiction with the results of the recent studies conducted in this field. For example, as mentioned in our study, only the feeling of weakness of the patients participating in the intervention group was significantly reduced compared to the control group, while in the other studies, most of the clinical symptoms such as weakness, muscle fatigue, cough, chill, sore throat, etc. have significantly decreased [8, 24, 37–39]. Regarding the inflammatory index of CRP, the results of prior studies were also contradictory. Some studies, such as Shafie et al., have reported consistent results with the present study and did not observe any significant effect of nano-curcumin supplementation on CRP levels [39], while clinical and systematic reviews, in this case, have reported positive effects of various forms of curcumin supplements on CRP levels [26, 37]. It should be noted that the proposed mechanism of studies observing the positive effects of curcumin supplementation on clinical signs and inflammatory markers has been stated that curcumin can have beneficial effects on gene expression and balance between pro-inflammatory (IL-6 and IL-1β) and anti-inflammatory factors (IL-10, IL-35, and TGF-α) and in this way, it can exert its effects on the control of this disease [26, 29, 40, 41]. Also, regarding the conflicting results of the present study and previous studies, we can point to factors such as differences in the type and dose of supplements used, as well as the duration of different studies. On the other hand, most other studies have been performed on hospitalized patients, while the present study has been performed on outpatients.

Excessive tiredness is a major complaint of COVID-19 patients, which very often remains for a long time, resulting in disability, and poor life quality [42]. This chronic fatigue syndrome can have long-lasting effects like debilitating exhaustion, pain, memory impairment, and sleep abnormalities [43, 44]. Currently, there is no medication to support fatigue and tiredness observed in COVID-19 patients. In this context, our study demonstrates the significant reduction in weakness and tiredness in patients supplemented with curcumin, suggesting its immense beneficial effect in log COVID.

Regarding the biochemical indices of WBCs, MCH, and MCHC, the values of the mentioned indices increased significantly in the placebo group after the end of the study. In the case of the WBCs, this significance was also observed marginally with the intervention group. The results of previous studies have shown a potential association between an increase in white blood cell count and severe COVID-19 [45]. A significant increase in MCH and MCHC [46] was observed in the placebo group, but the values were within the normal range. Therefore, considering the use of corticosteroids in the treatment process of COVID-19 and the increasing effect of these medicines on hemoglobin concentration, the results of the present study can be justified [47–49].

In the case of RDW, as reported in some animal and human studies, high doses of turmeric and curcumin can inhibit its absorption by binding to iron in the gastrointestinal tract and subsequently increase RDW levels [50–52]. Also, due to the prevalence of malnutrition among patients with COVID-19, this increase observed in RDW can be attributed to malnutrition of nutrients such as vitamin B12 [53–56].

The results of the present study showed that cholesterol levels in both intervention and control groups increased after the end of the study. The findings of the present study are in contradiction with the reducing effects of curcumin on cholesterol levels, the observed results of other COVID-19 studies, and the effects of inflammation and infection [57–60]. This discrepancy in results may be due to the small sample size, the short duration of the study, or/and the study performed on outpatients.

Since fatigue and weakness are some of the most common complications of COVID-19 disease, eliminating this complication can be of particular importance [61, 62]. The main finding of the present study is a significant reduction in weakness in the curcumin-piperine co-supplement group compared with the placebo group. COVID-19 disease leads to muscle wasting resulting in systemic inflammation, decreased physical activity, and lack of adequate nutrients, resulting in decreased muscle strength and a feeling of weakness [63, 64]. In previous studies, NF-kB has been mentioned as an effective factor and possible mechanism in muscle wasting through different catabolic situations [65–70]. As a result, by activity inhibition of NF-kB, curcumin can prevent muscle wasting and, consequently, the feeling of weakness caused by it. In a recent study, Powar et.al. observed that supplementation with curcumin and piperine could ensure early symptomatic recovery from fever, cough, sore throat, and breathlessness. They also found that curcumin supplementation could help in the maintenance of oxygen saturation above 94% without oxygen support, lesser deterioration, and better clinical outcomes compared to patients of the control group [71]. This study included mild, moderate, and severe COVID patients, while our study was restricted to patients with mild symptoms. In this study, most of the determining and important factors in this disease as well as the existing symptoms before and after the intervention have been investigated. However, there were limitations as follows: small sample size, study on outpatients, and sampling from one center were some of the limitations of the present study, therefore RCT studies with larger sample size, a higher dosage of curcumin-piperine and sampling of hospitalized patients, and different centers are recommended. Moreover, in this study, we did not consider biomarkers of the curcumin to evaluate the rate of adherence.

## Conclusion

The results of the present study showed that curcumin-piperine co-supplementation in outpatients with COVID-19 can significantly reduce weakness. However, in this study, curcumin-piperine co-supplementation could not significantly affect the other indices, including biochemical and clinical indices. More clinical trials with a larger sample size and higher dose and duration will be needed in the future to ascertain the use of curcumin as an adjunct therapy in COVID-19 patients.

## Data Availability

The data that support the findings of this study are available from the corresponding author, upon reasonable request.
